# Magnetic Performance and Anticorrosion Coating Stability of Thermally Demagnetized Nd-Fe-B Permanent Magnets for Reuse Applications

**DOI:** 10.3390/ma17235927

**Published:** 2024-12-04

**Authors:** Tomaž Tomše, Pierre Kubelka, Rosario Moreno López, Peter Fleissner, Laura Grau, Matej Zaplotnik, Carlo Burkhardt

**Affiliations:** 1 Institute for Precious and Technology Metals, Pforzheim University, 75175 Pforzheim, Germany; pierre.kubelka@hs-pforzheim.de (P.K.); rosariomoreno.bcn@gmail.com (R.M.L.); peter.fleissner@hs-pforzheim.de (P.F.); laura.grau@hs-pforzheim.de (L.G.); carlo.burkhardt@hs-pforzheim.de (C.B.); 2 Department of Nanostructured Materials, Jožef Stefan Institute, 1000 Ljubljana, Slovenia; 3 Jožef Stefan International Postgraduate School, 1000 Ljubljana, Slovenia; 4 Magneti Ljubljana, d.d., 1000 Ljubljana, Slovenia; matej.zaplotnik@magneti.si

**Keywords:** Nd-Fe-B magnets, thermal demagnetization, surface coating, microstructure, corrosion

## Abstract

Nd-Fe-B-type permanent magnets, containing approximately 30% critical rare-earth elements by weight, are essential components in renewable energy systems (e.g., wind turbines, hydroelectric generators) and electric vehicles. They are also critical for consumer electronics and electric motors in products like energy-efficient air conditioners and home appliances. In light of advancing sustainability goals, the direct reuse of magnets from end-of-life devices offers a promising alternative to energy-intensive and costly recycling methods based on hydro- and pyrometallurgical processes, as well as modern short-loop recycling through hydrogen processing. However, Nd-Fe-B magnets must be demagnetized before they can be extracted from devices. This study explores the effects of thermal demagnetization, performed either in air or a vacuum, on the stability of anticorrosion coatings and the magnetic performance of remagnetized magnets. Corrosion tests were conducted to assess the compatibility of various coatings with thermal demagnetization, identifying those most suitable for future applications involving magnet reuse.

## 1. Introduction

Nd-Fe-B-type permanent magnets are essential engineering materials used in numerous applications, including electric motors, wind turbines, consumer electronics, and home appliances [1]. The Critical Raw Materials Act (CRMA) has set an ambitious goal of recycling 25% of magnets by 2030 [2]. Long-loop recycling strategies based on conventional hydro- and pyrometallurgical approaches aim to extract and retain rare-earth elements (REEs) within the production cycle but are economically unsustainable [3,4]. The growing demand for advanced magnets has facilitated the introduction of new magnet-recycling paradigms, namely more energy-efficient and environmentally friendly short-loop recycling based on hydrogen processing of magnet scraps (HPMS). This allows for the recovery of the Nd-Fe-B alloy by embrittling and pulverizing end-of-life magnets, followed by further powder shaping and resintering [5,6].

Sintered Nd-Fe-B magnets are multiphase materials primarily based on the Nd_2_Fe_14_B hard magnetic phase. The secondary Nd-rich phases are prone to corrosion due to the very negative reduction potential of Nd (−2.32 V), which leads to the degradation of magnetic performance [7]. This issue is usually mitigated through the application of suitable anticorrosion coatings. However, the contamination of an HPMS-based powder with coating material residues could lead to downcycling [8]. An alternative to both long-loop and short-loop recycling is the direct reuse of magnets, provided that the Nd-Fe-B material has not been compromised by corrosion during the lifespan of the magnet-containing device. However, the magnets must be demagnetized before they can be separated and extracted from other components of the device. This is achieved by heating them above the Curie temperature (Tc), which is 312 °C for the pure Nd-Fe-B ternary system, but it can vary with chemical composition [9]. Thermal demagnetization is particularly effective when heating also breaks down the adhesive used to fix the magnets, such as in surface-mounted permanent-magnet synchronous motors.

This work aims to provide guidelines for future magnet design, specifically in selecting coating materials best suited for magnet reuse. We investigated the effect of heating on the magnetic performance of the remagnetized magnets and the stability of the coatings. Three types of sintered Nd-Fe-B magnets from different grades with common corrosion-protection coatings—Zn, Ni/Cu/Ni, and epoxy—were used as a case study. Niche coatings, such as Ni-Cu-Ni-Au, Ni-Cr, Ni-Sn, Al, and Al(Fe), were not included as they are rarely found on magnets. The cost-effective Zn-based metallic coatings act as a sacrificial anode, providing good corrosion resistance to chemically abrasive environments and low requirements towards the coating layer quality [10,11]. However, the low melting point of Zn at 420 °C poses a risk of diffusion into the Nd-Fe-B material at elevated temperatures [12]. Ni/Cu/Ni multilayer coatings are commonly found on end-of-life magnets. The intermediate Cu coating layer provides exceptional protection [13], while the multilayer structure prevents crack propagation [14]. Epoxy coatings are often used for their isolative nature and good chemical protection [10]. Epoxy resins, which are thermosetting polymers, undergo a curing process to form a rigid, cross-linked structure that can decompose at high temperatures [10,15].

Thermal demagnetization trials were conducted by heating the magnets in air or in a vacuum for 30 min at 380 °C, above their Tc values determined by differential scanning calorimetry (DSC). The results of microstructural analysis and magnetic characterization revealed that the choice of a coating material is important for reuse applications to prevent the degradation of magnetic performance.

## 2. Materials and Methods

### 2.1. Nd-Fe-B Magnets

Sintered Nd-Fe-B magnets, coated with different corrosion-protection coatings, were obtained from various sources. The geometries and dimensions of the magnets were as follows:

i.  Zn-coated—cylindrical: 11.0 (height) × 63.5 (diameter) mm.
ii.  Epoxy-coated—rectangular cuboids: 29.2 (length) × 6.5 (width) × 3.7 (height) mm.
iii.  Ni/Cu/Ni-coated—rectangular cuboids: 20.0 (length) × 6.0 (width) × 1.5 (height) mm.

### 2.2. Methods

Inductively coupled plasma–optical emission spectroscopy (ICP-OES) was used to determine the chemical compositions of the Zn-coated, epoxy-coated, and Ni/Cu/Ni-coated magnets. Approximately 0.1 g from each sample was digested in a solution of HCl and HNO_3_ in a CEM MARS 6 Microwave device. The digested samples were then diluted with 5% HNO_3_ and elemental analysis was performed with an ICP-OES iCap 7000 device from Thermo Scientific (Waltham, MA, USA). The protective coatings were removed before the analysis.

The Curie temperatures of the magnets were determined using differential scanning calorimetry (DSC) analysis. Approximately 100 mg of each sample was analyzed with a Netzsch STA 449C Jupiter device (Selb, Germany), which performs simultaneous thermogravimetric analyses. Measurements were conducted in air, heating the samples up to 1000 °C at a rate of 20 K/min, followed by cooling to room temperature.

Thermal demagnetization trials were performed by heating the magnets either in air or in a vacuum at 380 °C. The dwell time at this temperature was 30 min.

The second-quadrant demagnetization curves were obtained with a permeameter (Brockhaus Instruments Hystograph HG 200, Lüdenscheid, Germany). The epoxy-coated magnet was measured as a whole. For the Zn-coated magnet, a smaller rectangular piece was cut from the large cylindrical magnet. In both cases, measurements were conducted at room temperature. Three Ni/Cu/Ni-coated magnets were stacked together because their thickness was smaller than the measuring coil of the permeameter. In this case, the measurement was performed at 100 °C. The measurement uncertainty of the values obtained with the permeameter was less than 2%. Given the low thickness of the coatings, the resulting change in relative magnetic mass due to the coating is expected to fall within this uncertainty and is therefore not accounted for.

The microstructural and compositional analyses were performed with a field-emission gun–scanning electron microscope (FEG-SEM, FEI Nova NanoSEM 450, Hillsboro, OR, USA) equipped with an energy-dispersive X-ray (EDS) silicon drift detector (Oxford UltimMax 65, Wycombe, UK) at an acceleration voltage of 15 kV. The obtained mappings had a resolution of 2048 × 2048 pixels and a total acquisition time of 24 min, and the linescan had a length of 10 microns and a resolution of 50 points with an intermediate dwell time of 10 s per point. For the investigation, the initial magnets and thermally demagnetized samples were embedded in PolyFast, a carbon-based resin from Struers (Willich, Germany). They were then polished with SIC grinding papers, followed by 6 µm, 1 µm, and 0.25 µm waterless diamond suspension (Cloeren Technology, Wegberg, Germany).

Corrosion tests were performed on the initial coated magnets and thermally demagnetized samples. Salt-spray tests followed the ISO 9227 (NSS) standard [16]. Samples were placed on a plastic holder made from CR4 material according to ISO 3574 [17]. The holder was set at an angle of 20 degrees. A 5% NaCl solution with a pH range of 6.5–7.0 was atomized and sprayed over the samples for 120 h. The test was conducted at a constant temperature of 35 °C. Visual inspections of the samples were performed every 24 h to assess the extent of the corrosion. The procedure ensured a standardized and consistent evaluation of the corrosion resistance of the magnets under controlled conditions.

## 3. Results and Discussion

### 3.1. Chemical Composition and Magnetic Performance

The chemical composition of Nd-Fe-B-type magnets is often complex and carefully tailored to meet the requirements of specific magnet grades. The compositions of the three magnets—Zn-coated, Ni/Cu/Ni-coated, and epoxy-coated—were obtained using the ICP-OES technique after the removal of the protective coatings, as shown in Table 1. In addition to Nd, Fe, and B, other REEs, namely Pr, Dy, Tb, Ce, and Gd, are present in different amounts. Co improves the material’s high-temperature performance by increasing its Tc value [9]. Additional elements include Al, Cu, and Ga, which are often added for improved grain boundary wetting, which is required to magnetically decouple the Nd_2_Fe_14_B matrix grains in terms of exchange interactions [18]. Small amounts of Si, Zr, and Nb are included for grain refinement and enhanced thermal stability [18,19,20].

**Figure 1:**
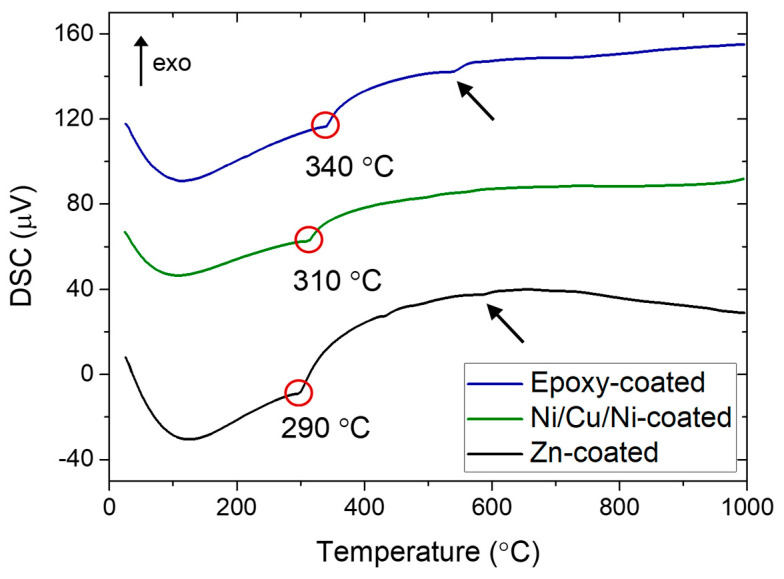
DSC curves of the magnets with marked Curie temperatures (red circles) and small endothermic peaks attributed to the melting of the rare-earth-rich phase (arrows). The coatings were removed before the analysis.

Figure 1 shows the DSC curves recorded for the magnets upon heating from room temperature to 1000 °C. The marked endothermic peaks (red circles) indicate the transition from the ferromagnetic to the paramagnetic state and correspond to the magnets’ respective Tc values. Arrows marking small endothermic peaks in the 550–600 °C range are attributed to the melting of the rare-earth-rich phase. The eutectic temperature of the ternary Nd-Fe-B system is ≈ 665 °C; however, alloying elements like Cu, Al, and Ga can reduce the melting temperature for complex Nd-Fe-B-type systems [18]. The lowest Tc value (290 °C) was measured for the Zn-coated magnet. Compared to the Curie temperature of the pure Nd_2_Fe_14_B phase (312 °C), this reduction is due to the addition of 3.13 wt.% of Ce and the lower Tc value of the Ce_2_Fe_14_B phase (151 °C) [21]. The Curie temperatures of the Ni/Cu/Ni and epoxy-coated magnets are 310 and 340 °C, respectively. The particularly high Tc value of the epoxy-coated magnet is attributed to the addition of 2.23 wt.% of Co and 11.39 wt.% of Dy (Table 1) due to the high Tc values of the Nd_2_Co_14_B and Dy_2_Fe_14_B phases (734 and 329 °C, respectively) [22,23]. Additionally, the Tc values of the Zn-coated and Ni/Cu/Ni-coated magnets are reduced by a significant amount of Pr (>7 wt.%) due to a slightly lower Tc value of the Pr_2_Fe_14_B phase (291 °C).

Based on the experimentally determined Curie temperatures, thermal demagnetization trials were performed at 380 °C with a 30 min dwell time. This temperature is 40 °C above the Curie temperature of the epoxy-coated magnet, ensuring complete demagnetization for all three magnets used in this study.

**Figure 2:**
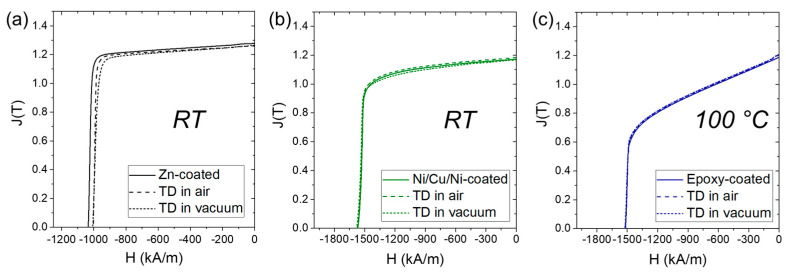
Second-quadrant demagnetization curves measured before and after thermal demagnetization (TD). (**a**) Zn-coated magnet measured at room temperature. (**b**) Ni/Cu/Ni-coated magnet measured at room temperature. (**c**) Epoxy-coated magnet measured at 100 °C.

Figure 2 shows the second-quadrant demagnetization curves measured for the magnet samples before and after thermal demagnetization. All the samples were magnetized to saturation before measurement. For the Zn-coated magnet (Figure 2a), a 3% drop in intrinsic coercivity (Hci) from 1035 to 1005 kA/m was measured for samples thermally demagnetized either in air or in a vacuum. Similarly, the remanence (Br) was reduced from 1.28 to 1.26 T, which falls within the measurement uncertainty. For the Ni/Cu/Ni-coated (Figure 2b) and epoxy-coated (Figure 2c) magnets, thermal demagnetization did not irreversibly deteriorate the magnetic performance, which was completely recovered upon remagnetization after heating. Of the three magnet samples, the epoxy-coated magnet exhibited the highest coercivity, which could not be quantified at room temperature due to insufficient maximum magnetic field provided by the permeameter. Its Hci value of 1520 kA/m at 100 °C is attributed to the high Dy content [24], amounting to 11.39 wt.% (Table 1). The specific chemical composition of this material, namely additions of Co and Dy, implies that the magnet was designed for high-temperature application.

### 3.2. Coating Stability Upon Thermal Demagnetization

**Figure 3:**
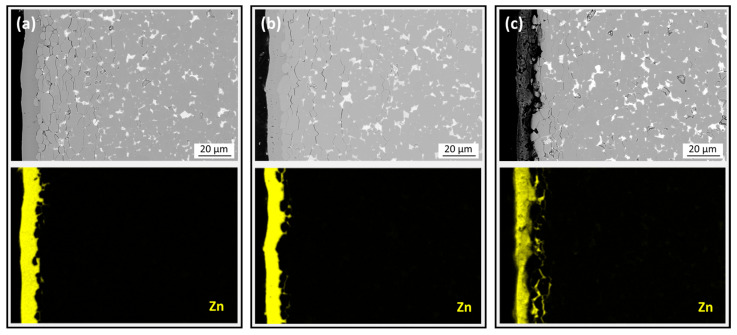
Backscattered-electron (BSE) mode SEM images of the polished cross-sections of Zn-coated samples with EDS mapping showing the distribution of Zn. (**a**) Before thermal demagnetization. (**b**) After thermal demagnetization in air. (**c**) After thermal demagnetization in a vacuum.

The effect of heating at 380 °C on the coating integrity of the Zn-coated magnet is shown in Figure 3. The backscattered-electron (BSE) mode SEM image of the cross-section near the edge of the initial magnet (Figure 3a) reveals a rough interface between the Nd-Fe-B material and the Zn coating, with clearly outlined surface grains. Cracks in the Nd-Fe-B close to the coating indicate damage during mechanical polishing prior to SEM analyses. The BSE imaging in a compositional contrast mode reveals the matrix phase (gray contrast) and Nd-rich phases (bright contrast). The Nd-Fe-B/Zn interface and coherence of the coating are further revealed through EDS mapping of the Zn distribution. It appears that the magnet was not polished before Zn coating to ensure good adhesion.

BSE-SEM images of the cross-sections of thermally demagnetized samples in air or vacuum are shown in Figure 3b,c, respectively. The Zn coating in Figure 3b appears intact, whereas the coating in Figure 3c is severely damaged. Since all samples were metallographically prepared for SEM analysis using the same procedure (Section 2.2), the damage to the coating’s integrity is attributed to the deteriorating effect of heating in a vacuum. The melting and boiling points of Zn (420 and 907 °C, respectively) are relatively low [25]. Consequently, Zn tends to have a higher vapor pressure at a given temperature compared to other metals with higher melting and boiling points. The SEM analyses indicate that the Zn coating can sublimate in vacuum.

**Figure 4:**
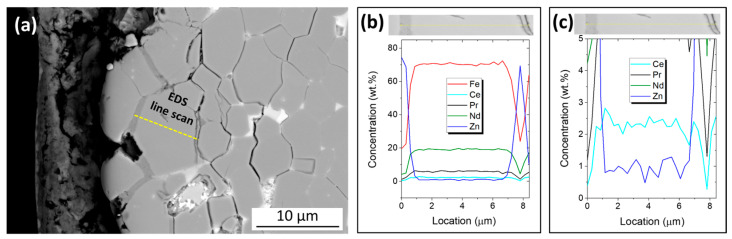
(**a**) Higher-magnification backscattered-electron (BSE) mode SEM image close to the Nd-Fe-B/Zn interface on a Zn-coated magnet after thermal demagnetization in a vacuum with a marked area of the EDS linescan (yellow line). (**b**,**c**) Elemental composition of a matrix grain and the surrounding phase determined via EDS linescan analysis at a higher and lower scaling, respectively.

As previously reported by Hu et al. [12], Zn can diffuse along the Nd_2_Fe_14_B grain boundaries at increased temperatures and form Fe-Zn grain boundary films surrounding the matrix grains. Figure 4a shows a higher-magnification BSE-SEM image taken near the edge region of a Zn-coated magnet after thermal demagnetization in a vacuum. An EDS linescan was performed to probe the chemical composition of a matrix grain and the surrounding phase, which displays a dark gray contrast. Analysis revealed that this phase is rich in Zn and Fe (Figure 4b), confirming that thermal demagnetization promotes the diffusion of Zn along the grain boundaries and the formation of Zn-enriched phases. As further shown in Figure 4c, Zn also diffused into the bulk of the matrix grain, with the Zn concentration in the grain ranging from 0.5 to 1.3 wt.%. The penetration depth of Zn into the Nd-Fe-B material and bulk diffusion in the matrix grains is likely limited to the region close to the Nd-Fe-B/Zn interface. However, changes in grain boundaries, phase composition, and phase distribution in the Nd-Fe-B magnets can adversely affect their magnetic properties [26]. The 3% decrease in coercivity after heating in air or in a vacuum (Figure 2a) is attributed to a compromised microstructure and, consequently, magnetically weakened surface regions of the magnet. Considering that the affected Nd-Fe-B material represents a relatively small part of the volume for the 11 mm thick Zn-coated magnets used in this study, the deterioration of coercivity and remanence is expected to be more pronounced for thinner magnets.

**Figure 5:**
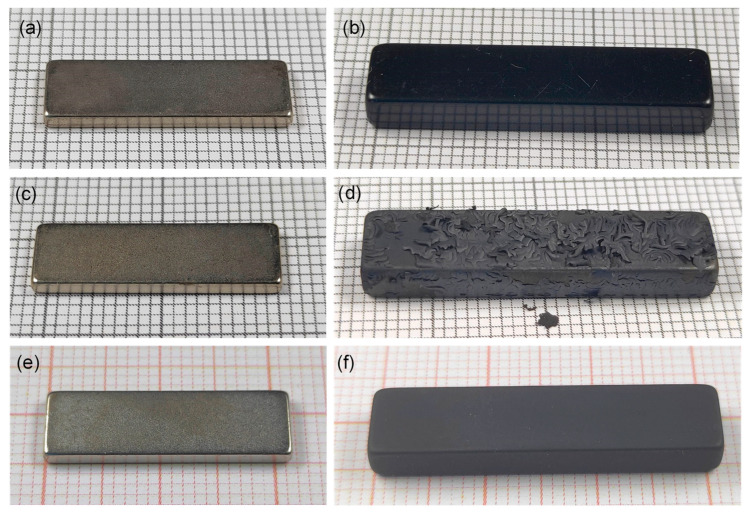
Photographs of the magnet samples on millimeter paper. Before thermal demagnetization: (**a**) Ni/Cu/Ni-coated and (**b**) epoxy-coated. After thermal demagnetization in air: (**c**) Ni/Cu/Ni-coated and (**d**) epoxy-coated. After thermal demagnetization in vacuum: (**e**) Ni/Cu/Ni-coated and (**f**) epoxy-coated.

For the Ni/Cu/Ni-coated and epoxy-coated magnets, whose magnetic performances were not irreversibly compromised by the thermal demagnetization, visual inspection of the samples was conducted before and after heating. Photographs of the samples are shown in Figure 5. For the Ni/Cu/Ni-coated magnet (Figure 5a), no impact of heating in air or in a vacuum on the coating was observed (Figure 5c,e, respectively). For the epoxy-coated magnet (Figure 5b), the coating decomposed and completely peeled off the Nd-Fe-B material during heating in air; the resulting flakes can be seen on the sample’s surface in Figure 5d. This behavior aligns with the reported decomposition temperature of epoxy resin in air, approximately 400 °C [27]. In contrast, upon heating in a vacuum, the coating did not peel off (Figure 5f); only a change in color from the glossy black of the original magnet to dull gray was observed.

**Figure 6:**
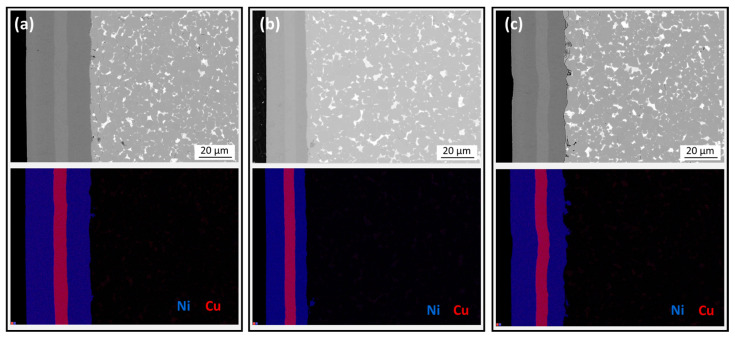
Backscattered-electron (BSE) mode SEM images of the polished cross-sections of the Ni/Cu/Ni-coated samples with EDS mapping showing the distribution of Ni and Cu. (**a**) Before thermal demagnetization. (**b**) After thermal demagnetization in air. (**c**) After thermal demagnetization in a vacuum.

Figure 6 shows BSE-SEM images taken of the cross-sections of the initial Ni/Cu/Ni-coated magnet (Figure 6a) and samples thermally demagnetized in air or in a vacuum (Figure 6b,c, respectively), along with the corresponding EDS mapping of Ni and Cu distribution. For all three samples, the EDS mapping showed chemically homogeneous Ni and Cu layers with no signs of interdiffusion between the coating layers and Nd-Fe-B. The coating thickness ranges between 23 and 40 µm, a pre-existing feature of the magnets that is not influenced by heating. The investigation revealed no effect of the thermal demagnetization, performed either in air or in a vacuum, on the Ni/Cu/Ni coating’s integrity.

**Figure 7:**
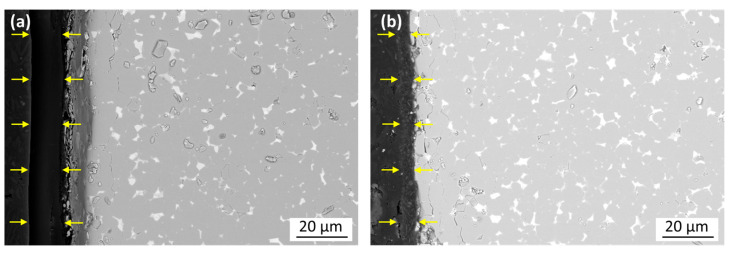
Backscattered-electron (BSE) mode SEM images of the polished cross-sections of the epoxy-coated magnet samples with arrows marking the epoxy layer. (**a**) Before thermal demagnetization. (**b**) After thermal demagnetization in a vacuum.

Figure 7 shows BSE-SEM images taken of the cross-sections of the initial epoxy-coated magnet (Figure 7a) and a sample thermally demagnetized in a vacuum that still retained its coating (Figure 7b). In Figure 7a, the interface between the epoxy and Nd-Fe-B is partially damaged, which is attributed to the polishing carried out before the analysis due to the difference in the mechanical properties of the two materials. In both cases, the coating is continuous, but it is significantly thinner in the thermally treated sample. Thermal demagnetization in a vacuum reduced the coating’s thickness from approximately 15 to 5 µm. This shrinkage is attributed to the pyrolysis, i.e., a partial fragmentation of the resin and the outgassing of volatile additives [15,28]. However, in the absence of oxidative reactions, the epoxy coating did not completely disintegrate and peel off, as was the case for heating in air.

### 3.3. Corrosion Protection

**Figure 8:**
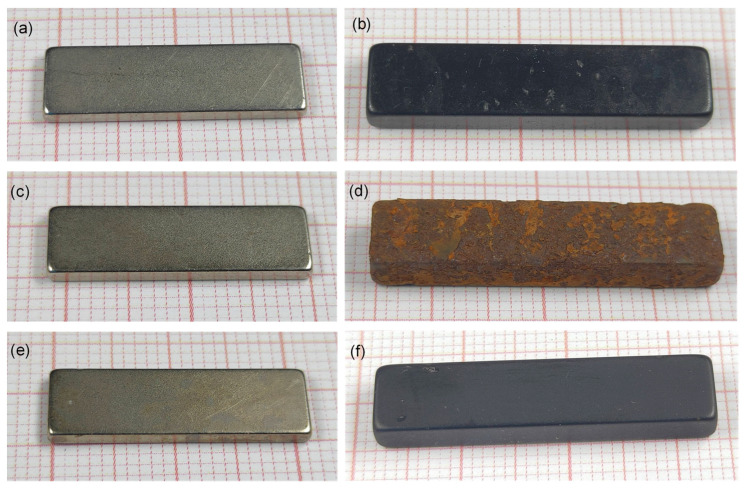
Photographs of the magnet samples on millimeter paper after corrosion tests. Before thermal demagnetization: (**a**) Ni/Cu/Ni-coated and (**b**) epoxy-coated. After thermal demagnetization in the air: (**c**) Ni/Cu/Ni-coated and (**d**) epoxy-coated. After thermal demagnetization in a vacuum: (**e**) Ni/Cu/Ni-coated and (**f**) epoxy-coated.

Corrosion tests were performed on the Ni/Cu/Ni-coated and epoxy-coated magnets, along with their corresponding thermally treated samples, to compare the corrosion protection before and after the thermal demagnetization. Photographs of the samples after the tests are shown in Figure 8. Upon visual inspection, no differences were observed for the Ni/Cu/Ni-coated samples between the initial magnet (Figure 8a) and the thermally demagnetized samples in air or in a vacuum (Figure 8c,e, respectively). The coating remained intact in all cases, with minor, random discoloration as the only observable effect of the corrosive environment.

The initial epoxy-coated magnet showed no signs of coating degradation or corrosion (Figure 8b). The sample without the protective coating, i.e., the originally epoxy-coated magnet thermally demagnetized in air, served as a control sample (Figure 8d). It exhibited severe corrosion, characterized by a rough surface and a red-brown color. In contrast, the epoxy-coated magnet thermally demagnetized in a vacuum performed well in the test. Its surface remained smooth and the sample appeared mostly intact, except for minor visible damages to the coating (Figure 8f). A major concern is the mechanical stability of the coating after heating. Partial degradation of the epoxy may compromise its mechanical strength, which poses a risk of damage. Indeed, the samples scratched easily after thermal demagnetization, suggesting that additional (re)coating of the magnets might be necessary to meet the requirements for magnet reuse. Nevertheless, these results show that the epoxy coating still offers good corrosion protection after heating the magnets in a vacuum at 380 °C for 30 min. This simplifies the extraction of the epoxy-coated magnets from a magnet-containing device by minimizing the risk of compromising the Nd-Fe-B material with impurities such as oxygen and carbon during heating.

## 4. Conclusions

Three types of corrosion-protection coatings—Zn, Ni/Cu/Ni, and epoxy—commonly used for sintered Nd-Fe-B magnets were investigated in terms of their stability upon thermal demagnetization at 380 °C for 30 min. This temperature exceeds the Curie temperature (Tc) of the RE_2_Fe_14_B phase in standard commercial Nd-Fe-B magnets and can be adjusted to achieve complete demagnetization for a specific magnet grade. Microstructural analysis and magnetic characterization revealed that heating promotes Zn diffusion along the Nd_2_Fe_14_B matrix grain boundaries and bulk diffusion into the grains, compromising the magnetic performance. A 3% drop in coercivity was confirmed for the 11 mm thick Zn-coated magnet. Given the volatile nature of Zn and its effect on magnetic performance, it is concluded that Zn coatings are not compatible with thermal demagnetization for direct magnet reuse. The epoxy coating completely decomposes when the magnet is heated in air, while heating in a vacuum preserves its corrosion-protection efficiency, albeit with a reduced coating thickness. Both Zn-coated and epoxy-coated magnets might be suitable for recoating after the removal of the compromised surface. Conversely, the Ni/Cu/Ni coating remains stable upon thermal demagnetization in both air and a vacuum, making these multilayer coatings most suitable for magnet reuse.

The results of this investigation confirm that the appropriate choice of coating material allows for magnets to be extracted from a device after thermal demagnetization without risking irreversible loss of magnetic performance or contamination of the Nd-Fe-B material with impurities. However, even if a suitable heating procedure can be implemented to thermally demagnetize the magnets, their recovery from a magnet-containing device is often not possible for technical reasons, such as the complex configuration of the device, or economic reasons, such as a low quantity of the magnetic material in different products (e.g., consumer electronics).

The future implementation of a suitable design for reuse of magnet-containing devices is needed to close the loop on the circular economy for critical rare-earth elements. Importantly, the reuse of magnets represents an attractive alternative to conventional long-loop recycling routes. Moreover, it overcomes the challenges associated with contemporary short-loop recycling strategies based on the HPMS process, i.e., (i) potential contamination of the Nd-Fe-B material with coating residues, and (ii) disruption of its primary chemical composition, which is usually carefully tailored to meet the requirements for a specific magnet grade, when mixing recycled powders from different sources.

## Figures and Tables

**Table 1:** Elemental composition (wt.%) of the magnets obtained by ICP-OES. The coatings were removed before analysis.

	Zn-Coated	Ni/Cu/Ni-Coated	Epoxy-Coated
Nd	20.53	21.95	20.53
Pr	7.24	7.46	0.06
Dy	0.11	1.40	11.39
Tb	/	/	0.08
Ce	3.13	/	/
Gd	0.49	0.55	/
Fe	65.74	64.48	63.80
Co	0.21	0.95	2.23
B	1.00	1.02	1.05
Al	0.81	1.31	0.30
Cu	0.21	0.16	0.18
Ga	0.05	0.29	0.00
Si	0.21	0.30	0.28
Zr	0.16	0.11	0.05
Nb	0.12	0.01	0.05

## Data Availability

The data presented in this study are available on request from the corresponding author. The data are not publicly available due to privacy reasons.
